# Changes in brain functional connectivity associated with transcutaneous auricular vagus nerve stimulation in healthy controls

**DOI:** 10.3389/fnhum.2025.1531123

**Published:** 2025-03-06

**Authors:** Daniel A. Monti, Nancy Wintering, Faezeh Vedaei, Alicia Steinmetz, Feroze B. Mohamed, Andrew B. Newberg

**Affiliations:** ^1^Marcus Institute of Integrative Health, Department of Integrative Medicine and Nutritional Sciences, Thomas Jefferson University, Philadelphia, PA, United States; ^2^Jefferson Integrated Magnetic Resonance Imaging Center, Department of Radiology, Thomas Jefferson University, Philadelphia, PA, United States

**Keywords:** transcutaneous auricular vagus nerve stimulation, fMRI, functional connectivity, cerebellum, amygdala, insula, frontal lobe, networks

## Abstract

**Purpose:**

A growing number of research studies have explored the potential effects of vagus nerve stimulation (VNS) on brain physiology as well as clinical effects particularly related to stress and anxiety. However, there currently are limited studies showing functional changes during different frequencies of stimulation and laterality effects transcutaneous auricular VNS (TaVNS). In this study, we evaluated whether TaVNS alters functional connectivity in the brain of healthy controls. We hypothesized that TaVNS would significantly alter connectivity in areas involved with emotional processing and regulation including the limbic areas, insula, frontal lobe regions, and cerebellum.

**Methods:**

We enrolled 50 healthy controls. Participants were placed in the MRI scanner with MRI compatible ear buds that provided TaVNS. Subjects underwent TaVNS in the left, right, and both ears in a randomized manner during the MRI session. Stimulation was provided for 5 min on and then there was a 5 min off period in between. To evaluate the primary outcome of neurophysiological effects, all participants received blood oxygen level dependent (BOLD) functional magnetic resonance imaging (fMRI) during the TaVNS on and off states.

**Results:**

The results demonstrated significant changes in functional connectivity during TaVNS that differed depending on the frequency of stimulation and which ear was stimulated. In general, areas of the brain that had altered functional connectivity included the frontoparietal regions, limbic regions, insula, and cerebellum. Interestingly, cognitive areas were also involved including parts of the temporal lobe, salience network, and default mode network.

**Conclusion:**

This study is an initial step toward understanding the functional connectivity changes associated with TaVNS. The findings indicate significant brain changes, particularly in areas that are involved with emotional processing and regulation, as well as cognition. Future studies can expand on this data and focus on specific patient populations to determine the effects of TaVNS.

## Introduction

Vagus nerve stimulation (VNS) is a rapidly expanding area of both treatment and research. It encompasses both invasive (implantable) and non-invasive (electro-simulative/physio-simulative) modalities such as transcutaneous auricular VNS (TaVNS). Non-invasive methods appear to be preferred in that they can be more readily modulated and do not bear the risks associated with surgical implantation of invasive systems, although they likely do not work for certain indications.

Studies have shown that VNS can reduce anxiety among patients suffering from an elevated state of arousal associated with PTSD (Wittbrodt et al., 2021). VNS is believed to trigger plasticity in brain areas such as the prefrontal cortex (PFC) and amygdala, increasing acetylcholine and reducing stress and anxiety (Hays et al., 2013). Furthermore, TaVNS has recently been shown to have effect in individuals suffering from various psychological conditions such as depression or anxiety (Ferstl et al., 2024). The potential advantages of TaVNS are: (1) it is designed specifically to address distressing stimuli and unresolved emotional memories; (2) it is a brief, time-limited intervention; and (3) its multi-modal design may appeal to and benefit a broader range of patients than a single mode intervention.

While there are a growing number of clinical studies exploring the potential benefit of TaVNS, there are few studies that have explored the neurophysiological effects. Furthermore, there are questions regarding optimal methods for using TaVNS which includes which ear to use and which frequency. While clinical trials could help explore such effects, neuroimaging can be beneficial in better determining how different TaVNS parameters might affect the brain. Such information could help guide future clinical trials.

The major goal of this study is to determine whether TaVNS alters brain function as measured by functional connectivity. Functional magnetic resonance imaging (fMRI) with Blood Oxygen Level Dependent (BOLD) sequences allows for the determination of functional connectivity between brain structures to assess how these structures interact with each other. Studies have demonstrated that many neurological and psychological conditions (e.g., concussion, anxiety, depression, etc.) are associated with changes in functional connectivity since the brain has been altered physiologically either as part of or in response to the disorder (Vedaei et al., 2021; Gallo et al., 2023). Furthermore, interventions designed to improve these conditions, either pharmacological or non-pharmacological, can alter functional connectivity as part of their therapeutic effect (Monti et al., 2018; Vedaei et al., 2024).

We hypothesized that TaVNS would specifically alter functional connectivity primarily in the areas of the brain involved in emotional processing and regulation. These areas would include frontal regions, limbic regions, the insula, and cerebellum. These areas have been implicated in a wide range of emotional processes and emotional disorders.

If changes in the brain’s functional connectivity can be observed during TaVNS, this can contribute to future studies designed to take advantage of the brain regions affected by TaVNS. Specifically, if it can be shown that brain regions involved in emotions and emotional processing, such as the limbic structures, frontal regions, insula, and cerebellum, are affected by TaVNS, these findings would help guide future clinical studies to explore its use in emotional disorders. Such disorders might include depression, anxiety, or PTSD.

## Methods

### Participants

Fifty healthy controls were enrolled from the local community and provided informed consent approved by the Institutional Review Board of Thomas Jefferson University. This study was also posted on clinicaltrials.gov (NCT05132881). Exclusion criteria included any history of major psychiatric disorder such as post-traumatic stress disorder, generalized anxiety disorder, major depressive disorder, and substance abuse or dependence. In addition, potential participants were excluded for use of psychotropic medications or current use of medications that would interfere with autonomic nervous system measures.

The subjects consisted of 50 participants, 13 men and 37 women with an average age of 36.9 ± 19.4 years. Once enrolled, subjects underwent the TaVNS fMRI scanning procedure as described below.

### VNS procedure

The VNS procedure was developed to be used in the MRI scanner. Subjects were placed in the MRI scanner with an earbud in each ear connected to a commercially available VNS device (Xen by Neuvana, Boca Raton, FL, USA). The TaVNS device sits within the ear canal in each subject according to the device specifications. The TaVNS device specifically applies stimulation to the posterior tragus and the posterior external auditory meatus, both of which are areas that have been shown to stimulate the vagus nerve as it passes near the ear (Badran et al., 2018; Bretherton et al., 2019). The stimulator enabled for each earbud to be used for VNS either separately or together. The intensity of the stimulation was adjusted for each subject until they reported mild discomfort and then it was reduced until they reported barely sensing the stimulation. In addition, the stimulator was able to deliver the stimulation with one of two frequencies (30 Hz or 100 Hz). The selection of the two frequencies of 30 Hz and 100 Hz was based on prior research that has focused substantially on these frequencies. While other frequencies could have been considered, our review of the current research suggests that 100 Hz TaVNS has been most associated with locus coeruleus and nucleus tractus solitarius effects (Sclocco et al., 2020; Yokota et al., 2022). In the research literature, 30 Hz TaVNS has been associated with wellbeing, neuroplasticity, and mood modulation (Bretherton et al., 2019) and has been particularly used in studies on depression and epilepsy (Kong et al., 2018).

Subjects were randomized to receive all of their respective stimulation with either 30 Hz or 100 Hz. The 25 subjects receiving 30 Hz stimulation were 11 male and 14 female. Mean age is 37.5 ± 19.2, 4 were left handed, mean height is 67.4 ± 3.7 inches, and mean weight is 161 ± 39 pounds. The 25 subjects receiving 100 Hz stimulation were 2 male and 23 female. Mean age is 36.4 ± 20.0, 3 were left handed, mean height is 65.7 ± 3.4 inches, and mean weight is 154 ± 33 pounds.

**Figure 1:**
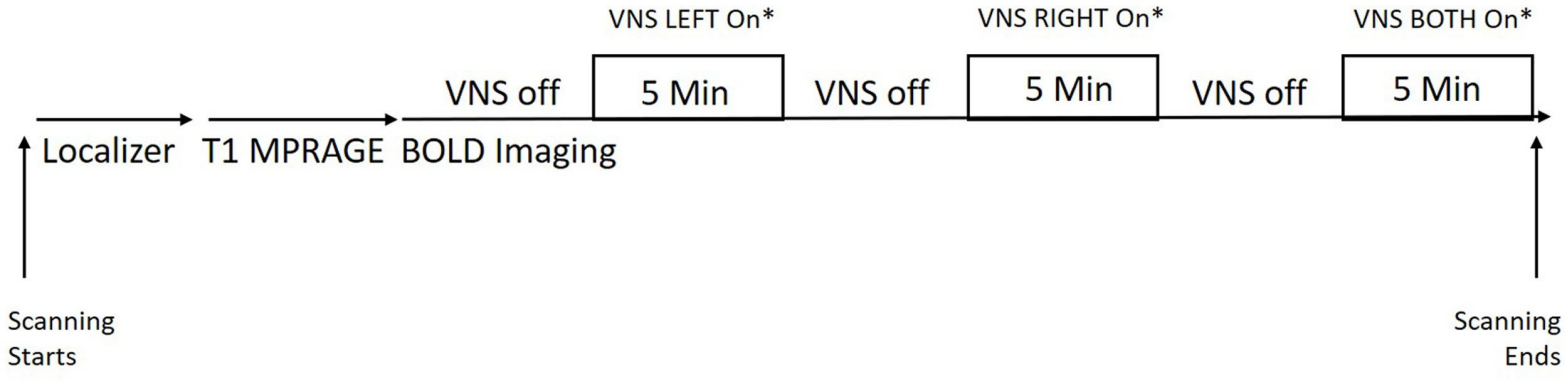
Scheme showing study set up with the TaVNS in the MRI scanner. *LEFT and RIGHT ordering was randomized within each subject. The stimulation frequency was randomized for each subject (subjects either received all stimulation at 30 Hz or 100 Hz).

While in the scanner, subjects underwent BOLD imaging throughout the time that the stimulator device was active. Subjects received LEFT or RIGHT ear stimulation first (in randomized order) and then BOTH ears stimulated in the final period. Each stimulation epoch lasted for 5 min with a 5 min off period in between (see Figure 1). It should be noted that subjects were not informed about whether they were randomized to LEFT or RIGHT first or to 30 Hz or 100 Hz.

### fMRI imaging protocol

fMRI data were obtained on all patients using a 3 T Siemens Biograph mMR PET-MR scanner with a 32-channel head coil. In order for further segmentation and registration steps during data pre-processing, an anatomical T1-image was obtained for all subjects. MRI parameters for the anatomical T1-weighted sequence were as follows: repetition time = 1.6 s, echo time = 2.46 ms, field of view = 250 × 250 mm, matrix = 512 × 512, voxel size = 0.49 × 0.49, 176 slices with slice thickness = 1 mm.

Next, BOLD scans were collected using the above described VNS paradigm with an Echo Planar Imaging (EPI) sequence to examine intrinsic FC of the brain regions. The following imaging parameters were used: FOV = 23.6 cm; voxel size = 3 × 3 × 4 mm^3^; TR = 2.0 s; TE = 30 ms; slice thickness = 4 mm; number of slices = 34; number of volumes = 300; and acquisition time = 600 s. During fMRI, the subjects were instructed to close their eyes, keep their heads still, and rest quietly without thinking about anything.

### Data processing

Data processing was performed using MATLAB-based programs, statistical parametric mapping^1^ and functional connectivity toolbox.^2^ The pre-processing was conducted using CONN default preprocessing pipeline. Initially, all the T1 structural data were oriented in the AC-PC line and their respective fMRI images reoriented to it using SPM12. All fMRI images were slice-timing corrected and realigned to the first volume using six-parameter rigid body transformation. The generated mean image was spatially normalized into standard stereotactic space, using the Montreal Neurological Institute (MNI) echo planar image (EPI) template. Computed transformation parameters were applied to all functional images, and the resulting images were smoothed using an 8-mm full-width half-maximum (FWHM), isotropic Gaussian kernel. In addition, artifact detection toolbox (ART) was set to the 97th percentile setting with the mean global-signal deviation threshold set at *z* = ±5 and the subject-motion threshold set at 0.9 mm. The artifact detection implemented in CONN was utilized to detect framewise displacement (FD). The computed motion parameters were then used to exclude the outliers. Any volumes which exceeded a motion threshold of 2 mm (translation) and 1° rotation, or more, were excluded. After preprocessing, by applying linear regression and a band-pass filter of 0.008–0.09 Hz, data were denoised to remove the effects of low and high frequency oscillations such as scanner drift, head motion, heart rate, and respiration rate. Then, the anatomical component-based, noise correction strategy (aCompCor) for spatial and temporal processing was used to remove non-neuronal noise factors from BOLD signal before computing connectivity measures. This method extracted principal components from white matter and cerebrospinal fluid (CSF) time series and used them as confounds during the denoising step (Behzadi et al., 2007). The implementation of aComCor along with the quantification of subject motion and the identification of outliers through (ART) allows for enhancement of specificity, sensitivity, and validity of first- and second-level connectivity analysis (Shirer et al., 2015).

### Functional connectivity analysis

After pre-processing steps, first-level and second-level functional connectivity analysis were conducted to generate ROI-based and Seed-based functional connectivity maps (Whitfield-Gabrieli and Nieto-Castanon, 2012). ROI-to-ROI analysis was performed by selecting a seed ROI, one by one, and the correlations of this seed with all other selected ROIs. The CONN toolbox provides predefined 164 ROIs composing an atlas of cortical and subcortical regions from the FSL Harvard-Oxford atlas, as well as cerebellar areas from the automated anatomical labeling (AAL) atlas. The atlas is normalized in MNI space and could be applied to the normalized data of the subject(s) (Rorden and Brett, 2000; Tzourio-Mazoyer et al., 2002). ROI-to-ROI analysis are Fisher *z*-transformed bivariate correlations between brain regions’ BOLD time-series that quantify associations in the activation at rest and serve as a proxy for connectivity. The time series were calculated by averaging voxel time series across all voxels within each ROI. For ROI-to-ROI analyses a threshold of *p* < 0.05 was used for bidirectional explorations of connectivity (i.e., positive and negative associations). Results of exploratory analyses were considered significant if they survived correction for multiple comparisons (*p*-FDR < 0.05). ROI-to-ROI analysis was executed separately for each condition of left, right, and bilateral stimulation each contrasted to no stimulation state fMRI data. Age, sex, and frequency used for the study were selected as the second-level covariates. Also, the same analysis was performed for subjects underwent stimulation with frequency 30 Hz and 100 Hz separately.

Further, seed-based functional connectivity was performed selecting brainstem since this region was found as the most common area with significant connectivity with other brain regions. So, the Fisher-transformed bivariate correlation coefficients between brainstem BOLD time series and each voxel BOLD timeseries were measured to generate brain functional connectivity maps. Cluster-level inferences on the between-group-level parametric statistics were based on false discovery rate multiple comparison correction with a voxel threshold *p* < 0.05 (uncorrected) and a cluster threshold *p* < 0.05 (cluster-size FDR corrected) for bidirectional explorations of connectivity (i.e., positive and negative associations). Seed-to-voxel connectivity was performed for each condition of left, right, and bilateral stimulation separately that each contrasted to no stimulation state fMRI data. In the next step, connectivity maps were generated for subjects underwent frequency 30 Hz and 100 Hz separately.

## Results

**Figure 2:**
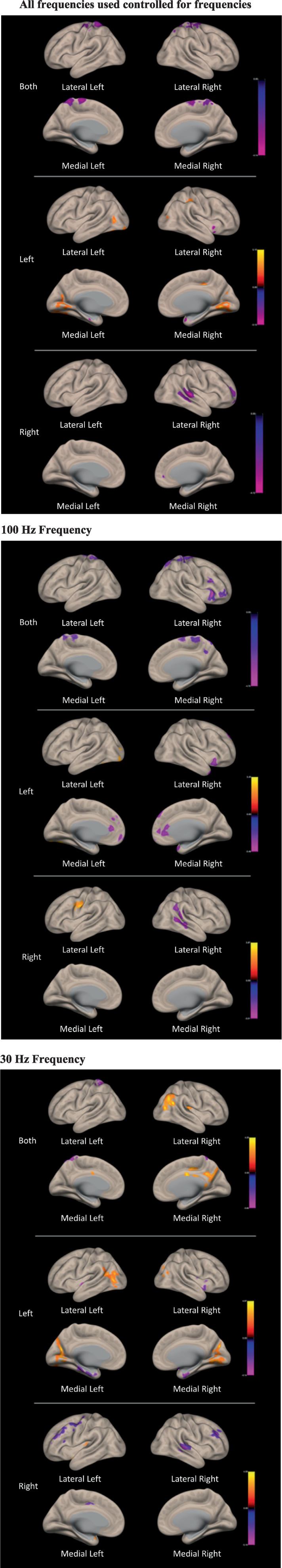
Figures for the functional connectivity changes associated with VNS when using the brainstem seed based analysis. There are three panels showing the changes associated with both frequencies on, and then the 100 Hz and the 30 Hz VNS.

The ROI-to-ROI functional connectivity results showed a number of significant changes associated with the use of the TaVNS (see Tables 1, 2). The changes were distinguished between the left, right, and bilateral stimulation. Further, the changes were distinguished based on the frequency of the stimulation. The seed based analysis of functional connectivity using the brainstem as representative of the locus ceruleus is shown in Figure 2 and Table 3.

**Table 1:** ROI-to-ROI analysis TaVNS on vs off (for both frequencies) with significant functional connectivity between the Region 1 and Region 2, *p*-FDR < 0.05.

Region 1	Region 2	*T*-value	*p*-FDR corrected
BOTH on vs. off			
Cerebellum L	Supracalcarine Cortex L	3.96	0.038
Cerebellum L	Intracalcarine Cortex L	3.70	0.038
Cerebellum L	Visual Medial Networks	3.57	0.038
Cerebellum L	Intracalcarine Cortex R	3.54	0.038
Cerebellum L	Supracalcarine Cortex R	3.39	0.045
Cerebellum L	Cuneal L	3.35	0.045
LEFT on vs. off			
Cerebellum L	Anterior Middle Temporal Gyrus R	−4.24	0.018
Middle Temporal Gyrus L	Supracalcarine Cortex L	5.31	0.0005
Middle Temporal Gyrus L	Supracalcarine Cortex R	3.71	0.046
Salience Networks_Supramarginal Gyrus	Superior SensoriMotor Network	−4.00	0.037
FrontoParietal Networks L	Default Mode Network	3.87	0.03
FrontoParietal Networks L	Precuneus	3.84	0.03
FrontoParietal Networks L	Frontal Medial Cortex	3.65	0.036
RIGHT on vs. off			
Cerebellum R	Accumbens L	−4.17	0.022
Inferior Lateral Occipital Gyrus R	Precentral Gyrus R	−3.79	0.047
Inferior Lateral Occipital Gyrus R	Salience Network Anterior Insula R	−3.65	0.047
Inferior Lateral Occipital Gyrus R	Planum Polare R	−3.65	0.047
Inferior Lateral Occipital Gyrus R	Planum Polare L	−3.47	0.047
Posterior Inferior Temporal Gyrus L	Posterior Supramarginal Gyrus R	−3.99	0.039
Subcallosal Cortex	Heschl’s Gyrus L	−4.14	0.024

**Table 2:** ROI-to-ROI analysis TaVNS on vs. off depending on frequency used.

Region 1	Region 2	*T*-value	*p*-FDR corrected
BOTH on vs. off Frequency 100 Hz			
Caudate R	Precentral Gyrus R	4.36	0.037
Temporal Occipital Fusiform Cortex L	Precentral Gyrus R	−4.34	0.02
Temporal Occipital Fusiform Cortex L	SensoriMotor Networks_Lateral R	−4.32	0.02
Temporal Occipital Fusiform Cortex L	Central Opercular R	−4.06	0.02
Temporal Occipital Fusiform Cortex L	Parietal Operculum R	−3.79	0.03
Temporal Occipital Fusiform Cortex L	Salience Networks_Supramarginal Gyrus	−3.76	0.03
BOTH on vs. off Frequency 30 Hz			
Posterior Middle Temporal Gyrus R	Hippocampus R	4.34	0.03
Postcentral Gyrus L	Inferior Lateral Occipital Gyrus R	−4.85	0.009
Parietal Operculum R	Cuneal L	−4.22	0.048
Cerebellum L	Posterior Supramarginal Gyrus L	−4.48	0.025
Planum Temporale R	Vermis	4.35	0.035
LEFT on vs. off Frequency 100 Hz			
Language Networks_Inferior Frontal Gyrus	Inferior Temporal Gyrus R	4.69	0.02
Frontal Pole L	Insular Cortex R	5.80	0.001
Vermis	Inferior Frontal Gyrus Operculum L	5.43	0.003
Vermis	Dorsal Attention Networks. Intraparietal Sulcus R	3.93	0.042
Vermis	Anterior Supramarginal Gyrus R	3.93	0.042
LEFT on vs. off Frequency 30 Hz			
Anterior Superior Temporal Gyrus L	Superior Frontal Gyrus R	4.78	0.0097
Anterior Superior Temporal Gyrus L	Superior Frontal Gyrus L	4.66	0.0097
Anterior Superior Temporal Gyrus L	Angular Gyrus L	4.04	0.029
Cerebellum R	Visual Medial Networks	4.05	0.035
Cerebellum R	Angular Gyrus R	−3.97	0.035
Cerebellum R	Occipital Fusiform Gyrus L	3.88	0.035
Cerebellum R	Cuneal L	3.84	0.035
Cerebellum R	Caudate L	−3.65	0.04
Cerebellum R	Intracalcarine Cortex R	3.63	0.04
Caudate L	Lingual Gyrus L	−4.80	0.014
Caudate L	Lingual Gyrus R	−4.39	0.018
Caudate L	Intracalcarine Cortex R	−4.22	0.019
Caudate L	Visual Medial Networks	−4.06	0.02
Salience Networks_Supramarginal Gyrus	Salience Networks_Anterior Insula L	−4.32	0.045
Salience Networks_ Prefrontal Cortex R	Supracalcarine Cortex R	4.24	0.027
Salience Networks_ Prefrontal Cortex R	Supracalcarine Cortex R	4.17	0.027
Salience Networks_ Prefrontal Cortex R	Supracalcarine Cortex L	4.07	0.027
Anterior Middle Temporal Gyrus L	Cerebellum L	−4.47	0.031
RIGHT on vs. off Frequency 100 Hz			
Cerebellum R	Angular Gyrus L	−4.62	0.024
Cerebellum R	Angular Gyrus R	−4.18	0.034
Hippocampus L	Posterior Cingulate Gyrus	−4.69	0.02
Planum Polare R	Dorsal Attention Networks_Inferior Parietal Sulcus R	−4.61	0.024
Anterior Inferior Temporal Gyrus L	Lingual Gyrus R	4.64	0.023
Anterior Superior Temporal Gyrus L	Inferior Temporal Gyrus R	4.71	0.019
Dorsal Attention Networks. Inferior Parietal Sulcus R	Insular Cortex R	−4.05	0.046
RIGHT on vs. off Frequency 30 Hz			
Vermis	Occipital Pole R	−4.76	0.015
Vermis	Occipital Fusiform Gyrus R	−4.42	0.017
Vermis	Salience Networks_Rostral Prefrontal Cortex L	4.14	0.023
Planum Polare R	Posterior Supramarginal Gyrus L	4.30	0.033
Planum Polare R	Inferior Lateral Occipital Cortex L	4.15	0.033
Planum Polare R	Superior Parietal Lobule R	3.91	0.04
Planum Polare R	Visual Lateral Networks L	3.77	0.043
Posterior Supramarginal Gyrus L	Amygdala R	5.34	0.003
Supplementary Motor Area L	Superior Parietal Lobule L	6.05	0.0007

**Table 3:** Results from the seed-based analysis measuring the brainstem seed-to-voxel FC during TaVNS, *p* < 0.05 voxel and *p*-FDR < 0.05 at cluster level.

Clusters (*x*, *y*, *z*)	Region BOTH vs. OFF	Size	Size *p*-FDR
−24, −40− +64	Postcentral Gyrus Left Precentral Gyrus Left	2,570	<0.0001
	**Region BOTH vs. OFF, Frequency 100 Hz**		
14, −60, +54	Postcentral Gyrus Right Precentral Gyrus Right	1,349	0.0017
24, +34, +0	Frontal Pole Right Frontal Orbital Cortex Right	1,100	0.0039
	**Region BOTH vs. OFF, Frequency 30 Hz**		
36, −64, +40	Lateral Occipital Cortex Right Angular Gyrus Right	1,259	0.0008
14, −18, +46	Precuneous Cortex Cingulate Gyrus, posterior division	1,202	0.0008
−24, −46, +64	Postcentral Gyrus Left Superior Parietal Lobule Left	791	0.0113
	**Region LEFT vs. OFF**		
10, −64, −2	Lingual Gyrus Right Occipital Fusiform Gyrus Left	4,056	<0.0001
−8, −18, −28	Putamen Right	1,134	0.0013
−8, −18, −28	Postcentral Gyrus Right Supramarginal Gyrus Right	697	0.0239
	**Region LEFT vs. OFF, Frequency 100 Hz**		
−52, −50, −30	Occipital Fusiform Gyrus Left	1,306	0.0014
2, +50, +2	Cingulate Gyrus, anterior division	1,155	0.0019
22, +22, +2	Temporal Pole Right Caudate Left	1,005	0.0035
	**Region LEFT vs. OFF, Frequency 30 Hz**		
−16, −66, +0	Lingual Gyrus Right Intracalcarine Cortex Right	2,340	<0.0001
12, −24, −22	Parahippocampal Gyrus, anterior division Left	1,344	0.0001
22, +4, −10	Parahippocampal Gyrus, anterior division Right Insular Cortex Right	1,183	0.0003
−44, −52, +18	Lateral Occipital Cortex Left	876	0.0022
−40, −70, −22	Cerebellum Left	592	0.0203
	**Region RIGHT vs. OFF**		
60, −42, +6	Middle Temporal Gyrus Right	979	0.0148
22, +48, +8	Frontal Pole Right	744	0.0409
	**Region RIGHT vs. OFF, Frequency 100 Hz**		
60, −42, +6	Angular Gyrus Right Middle Temporal Gyrus Right	1,104	0.0064
−48, −2, +30	Precentral Gyrus Left Postcentral Gyrus Left	776	0.0320
	**Region RIGHT vs. OFF, Frequency 30 Hz**		
−38, +4, +64	Precentral Gyrus Left Middle Frontal Gyrus Left	741	0.0287
−44, +42, +28	Middle Frontal Gyrus Left Frontal Pole Left	730	0.0287
30, +34, +30	Frontal Pole Right Middle Frontal Gyrus Right	653	0.0358
−20, −12, −10	Amygdala Left	562	0.0481
64, −26, +0	Superior Temporal Gyrus, posterior division Right Middle Temporal Gyrus, posterior division Right	558	0.0481

## Discussion

Overall, the TaVNS stimulation in this study resulted in altered functional connectivity between a number of important brain structures. The cerebellum had changes in functional connectivity with the visual areas. When the left vagus nerve was stimulated, there were changes in connectivity between the cerebellum and middle temporal gyrus, and the middle temporal gyrus and visual areas. There were also significant changes between the salience network and sensorimotor area as well as the frontoparietal network and the DMN, precuneus, and medial frontal cortex. When the right vagus nerve was stimulated, there were changes in connectivity between the cerebellum and nucleus accumbens, and between the visual areas of the occipital lobe and the salience network, precentral gyrus, and planum polare in the temporal lobe.

These findings are consistent with other studies evaluating TaVNS with fMRI which showed altered activity in the postcentral gyrus, insula, frontal cortex, right operculum, cingulate gyrus, and cerebellum (Badran et al., 2018). The insular connection with the frontal regions, particularly the medial PFC, has been found to be a focus of effect of TaVNS (Zhang et al., 2024). Other studies have found changes in limbic structures, temporal regions, thalamus, nucleus accumbens, and basal gangla (Dietrich et al., 2008; Kraus et al., 2013; Frangos et al., 2015; Yakunina et al., 2017). Such findings are consistent with our current study demonstrating changes in functional connectivity affecting most of these structures (see additional details below).

Additionally, EEG studies of TaVNS have yielded similar results to our current study (Gianlorenco et al., 2022). For example, an EEG study by Konakoğlu et al. (2023) showed that left-unilateral and right-unilateral auricular VNS causes delta and theta increase in the frontal regions, a finding supported by the current study showing important functional connectivity changes affecting the frontal regions. Another study of heart evoked potentials further implicated brain regions such as the orbitofrontal cortex, postcentral gyrus, precentral gyrus, insula, middle frontal gyrus, and temporal regions affected by TaVNS (Poppa et al., 2022).

There were distinctions between functional connectivity changes depending on the frequency of the stimulation as well. The 30 Hz frequency revealed changes in the prefrontal cortex, hippocampus, temporal lobe, visual regions, and cerebellum. When the left vagus nerve was stimulated at 30 Hz, there were a number of changes involving the superior temporal lobe, cerebellum, angular gyrus, visual regions, caudate nucleus, and salience networks. When the right vagus nerve was stimulated at 30 Hz, there were changes involving the vermis, planum polare, salience networks, visual areas and amygdala.

The 100 Hz frequency TaVNS revealed changes particularly in the occipital-fusiform region and the precentral gyrus, sensorimotor regions, and salience network. Left TaVNS at 100 Hz revealed changes in the vermis and dorsal attention networks, while stimulation of the right vagus nerve resulted in changes involving the cerebellum, hippocampus, planum polare, dorsal attention network, and insula.

These differences in functional connectivity effects between the two frequencies respectively, and when analyzed together suggests some overlap and some distinctions. Thus, the results from the combined analysis suggests the overall effects of TaVNS, while the distinction between the two frequencies or the two sides suggests more specific changes. These more specific changes can become diluted or amplified when the entire group is evaluated.

Finally, since it is known that VNS is supposed to involve the locus ceruleus in the brainstem, we applied a specific seed based analysis to determine if there were any significant changes between this region and other brain regions. The results revealed altered functional connectivity with the precentral and postcentral gyrus and supplementary motor areas. There were distinctions between the left and right stimulation with the left involving changes in the visual cortex, cerebellum, basal ganglia, cingulate gyrus, frontal regions, and parahippocampus. Stimulation of the right vagus nerve was associated with changes to the temporal lobe, supramarginal gyrus, precentral and postcentral gyrus, basal ganglia, and amygdala. As with the general analysis, there were also subtle distinctions in functional connectivity between the brain stem and other brain regions depending on the frequency involved. Taken together, these findings suggest a possible neurobiological basis for regulating stress and anxiety through TaVNS that likely involves the locus ceruleus, particularly with regard to its functional connectivity with areas that regulate emotions.

From a therapeutic perspective, several studies have found that stimulation of the vagus nerve not only has clinical benefits, but alters functional connectivity in areas similar to those observed in the present study. For example, one study in patients with migraine showed TaVNS was associated with brain stem regions of the vagus nerve pathway and brain regions associated with the limbic system, pain processing areas such as the postcentral gyrus, thalamus, and PFC, and basal ganglia (Huang et al., 2023).

The results from our VNS study also suggest a laterality effect depending on which side of the vagus nerve is stimulated. This is consistent with previous clinical studies which have also suggested a distinction between left and right vagus nerve functions. Studies comparing left-sided and right-sided taVNS in patients with seizures have shown mixed results. Some studies suggest that left-sided stimulation is more effective in reducing seizure frequency, possibly due to its more direct influence on the left hemisphere, which is often the dominant hemisphere for controlling epileptic activity (Bauer et al., 2016). However, other research indicates that right-sided taVNS may be equally effective or could provide additional benefits in certain patients, such as those with right-hemispheric seizure foci or bilateral seizure activity (Yuan and Silberstein, 2016).

Additionally, individual differences in vagal nerve anatomy and brain connectivity could influence the response to taVNS, making personalized approaches to stimulation site selection important. The left vagus nerve may be particularly useful in patients with cognitive impairment, altering functional connectivity in structures such as the left hippocampus, left temporal regions, and salience networks (Murphy et al., 2023). We found similar alterations in left sided functional connectivity in this group of healthy individuals with left TaVNS. Stimulation of the right vagus nerve may be more effective for conditions involving motor activity or visuo-spatial processing.

We had hypothesized and found that areas affected by TaVNS in this study appear to be associated with emotional regulation and processing. This includes areas such as the insula and limbic regions. Altered functional connectivity associated with VNS stimulation may help understand some of the therapeutic trials in which the stimulation has improved anxiety, stress, and depression. For example, studies of depression have found that TaVNS was associated with alterations in functional connectivity in the precuneus and middle frontal gyrus, and the left posterior cingulate gyrus and the left angular gyrus (Sun et al., 2023). Future studies should explore the differential effects of left versus right versus bilateral stimulation of the vagus nerve in various therapeutic settings.

Another important area that appears to be associated with TaVNS in the present study is the cerebellum. Such an effect could have several clinical consequences. The cerebellum has long been considered the brain structure involved in motor coordination. Several studies have found that VNS can help improve motor coordination in stroke patients, especially when combined with physical therapy (Korupolu et al., 2024). Our finding of changes in functional connectivity, especially with respect to motor areas, may help provide an underlying neurobiological basis of this clinical finding.

In addition, recent research by our team, and others has found that the cerebellum is involved in the mediation of intense emotional reactions. The cerebellum may be integral to the experience of emotions and the development of emotional memories (Baumann and Mattingley, 2012). In fact, distinct subregions of the cerebellum are believed to be related to negative emotional processing (Park et al., 2008; Ferrucci et al., 2012). The potential role of the cerebellum in modulating emotions and autonomic reactivity has been supported by clinical and neuroimaging data (Stoodley and Schmahmann, 2009, 2010). The implication would be that stimulation of the vagus nerve, subsequently affects cerebellar function, and helps with emotional regulation.

Of particular relevance to the present study, prior fMRI studies show that negative emotional stimuli activate the cerebellum, posterior cingulate, and fusiform gyrus (Park et al., 2008; Schraa-Tam et al., 2012). In addition, reciprocal connections link the cerebellum with brainstem areas containing neurotransmitters involved in mood regulation, including serotonin, norepinephrine, and dopamine (Dempesy et al., 1983; Marcinkiewicz et al., 1989).

The cerebellum connects with the limbic structures both ipsilaterally and contralaterally (Cacciola et al., 2017). It has also been found that the vermis may be particularly connected to the limbic structures (Blatt et al., 2013). Using MRI techniques similar to the current study, several resting state functional connectivity studies have found functional coherence between the cerebellum and amygdala, hippocampus, hypothalamus, insula, and anterior cingulate (Seeley et al., 2007; Sang et al., 2012; Allen et al., 2005). Neuroimaging studies suggest the cerebellum is associated with emotional circuits such that positive emotions are associated with the left cerebral hemisphere and negative emotions are associated with the right hemisphere (Silberman and Weingartner, 1986; Lee et al., 2004). Finally, our research has found significant changes in the cerebellar functional connectivity when patients with traumatic memories were treated with a mind–body intervention (Monti et al., 2018). Thus, the finding of cerebellar effects resulting from TaVNS in the present study supports its potential use in patients trying to manage various traumatic events and emotions.

In addition to the laterality observed on the present study, we explored the effect of two different frequencies of stimulation. It has been hypothesized that altering the frequency of VNS may affect the overall physiological response as well as potential clinical effects. In our study, we found two different frequencies, one at 30 Hz and the other at 100 Hz, resulted in distinct changes in functional connectivity. These distinctions might also inform future therapeutic studies to try to affect areas most relevant for given conditions.

Our findings are consistent with the findings from previous studies utilizing different frequencies. For example, studies using 25 Hz frequency for TaVNS have found activation in areas of the brain responsible for cognitive & emotional processing and balance and GABAergic neuromodulation (Keute et al., 2018; Badran et al., 2018). The use of 30 Hz for TaVNS has been found to improve age-related autonomic, mood, and sleep changes, and reduce fatigue (Clancy et al., 2014; Bretherton et al., 2019; Aranow et al., 2021). TaVNS using a frequency of 100 Hz has been found to activate brainstem nuclei responsible for pain, learning, memory, and clinically has been shown to support healthy blood pressure, especially when combined with breathwork (Sclocco et al., 2020; Yokota et al., 2022).

This study is one of the largest we are aware of that uses fMRI to measure the effects of TaVNS. This larger sample size enabled us to explore laterality of vagus nerve simulation, as well as the effects of differential frequencies of simulation. Several limitations should be considered when evaluating the data. The BOLD imaging paradigm used was relatively brief in order to accomplish all of the imaging within a one-hour session. However, our prior research studies have suggested that this timeframe is able to provide significant findings, which is why this particular protocol was used.

In addition, since we were interested in observing stimulation of the left, right, and both sides, we included all three types of stimulation during the single imaging session with the left and right performed in a randomized order. We kept a 5 min off period between the stimulation periods to allow for a wash-out of any effects. However, it is not clear how long the VNS effect might last which could complicate interpretation of the findings. There are limited studies on the duration of the VNS effect when turned on for relatively short periods of time. For example, research on heart rate variability indicates that TaVNS induces immediate parasympathetic activation that is short-lived, occurring only during stimulation and not persisting after it stops (Keute et al., 2018). However, a study of TaVNS evaluated the P300 cognitive event-related potential (ERP) as an indirect marker that reflects NE brain activation and found persistent effects up to 28 min after stimulation for 7 min (Gurtubay et al., 2023). Future studies might explore a potential washout of the VNS effect and also include longer off periods between stimulations. This could be performed with longer off periods within a single imaging session, or with imaging on separate days. In addition, fMRI could be performed at several time points (e.g., 10 min, 30 min, and 60 min) after a single stimulation in order to determine how long the changes in functional connectivity persist. Another technical limitation was the selection of the frequency of TaVNS for the present study. As mentioned, we selected 30 Hz and 100 Hz based on existing literature suggesting these frequencies to be particularly effective both physiologically and clinically. However, future studies can expand upon the present data by exploring a broader range of frequencies to help determine those that produce the greatest effect on functional connectivity.

All subjects were healthy individuals, and while the subjects were randomized, it turned out that the group receiving 30 Hz stimulation had substantially more males compared to the group that received 100 Hz. It seems unlikely that such a difference in the group demographics would be responsible for the differences in the findings between the two stimulation frequencies. However, future studies might include larger populations to ascertain any differences in responses based on age, gender, or other factors, as well as whether the changes observed would be comparable in various patient populations. Thus, similar studies should be considered for patients with stroke, anxiety, and depression. Finally, future studies might also consider adding additional comparison groups such as a sham stimulation, in which the stimulation takes place, but at different locations. Such an approach could improve the accuracy of the findings and also could help identify potential side effects such as sensing the stimulation or stimulation artifacts. Any side effects could then be potentially eliminated from the evaluation of the real VNS.

## Conclusion

Overall, the findings in this paper suggest that TaVNS appears to be associated with a number of changes in functional connectivity between brain structures. Several specific connections between cortical and limbic areas; cerebellum and limbic areas; and the brainstem and limbic areas; indicate important changes associated with TaVNS stimulation. These findings helped to demonstrate the neurobiological effects of TaVNS, but also generate hypotheses for future clinical trials, focusing on a variety of cognitive and emotional processes that might be addressed through TaVNS.

## Data Availability

The raw data supporting the conclusions of this article will be made available by the authors without undue reservation.

## Footnotes

^1^SPM12, https://www.fil.ion.ucl.ac.uk/spm/.

^2^CONN22a, https://web.conn-toolbox.org/.
